# Supplementary material for: The impact of differences in text segmentation on the automated quantitative evaluation of song-lyrics
Source: PLoS One. 2020 Nov 9;15(11):e0241979. doi: 10.1371/journal.pone.0241979 (PMC7652311; doi:10.1371/journal.pone.0241979)
Supplement: Attachment — Submitted filename: Response_to_Reviewers.pdf [file pone.0241979.s003.pdf]

September 10, 2020

Response to Reviewers
by
Dr. Katharina Parry
Dr. Friedrike Tegge

Dear Reviewers,

as a general comment we want to express our sincere gratitude to you both for taking the time to provide feedback on our work. These are difficult times so we are very appreciative of your efforts, thank-you.

Response to reviewer 1:

Original comment: *The study is well conducted, with a clear and precise analysis. The only slight drawback is the relatively small size of the sample considered (30 songs in total) and the relatively small number of annotators involved (3). If the conclusions drawn are valid, it would have been nice to obtain them on a larger set of songs and annotators.*

Thank you for pointing this out. We have added the following two paragraphs:

1. In the Methods section: "We note that the sample of 30 songs and three annotators is small. The work presented here is a first step in a larger project assessing the readability of pop songs for ESL learners. It is intended to address research-methodological problems that seem to be highly relevant not only to the wider investigation at hand but also to the use of Coh-Metrix and automated text evaluation more widely."
2. At the end of the Discussion section: "While the results of the present study point to the possibility of subjective bias being introduced in automated language evaluation due to punctuation, we note that the sample of 30 songs and only three punctuators is small and presents a limitation. Our work so far represents a preliminary study, and a follow-up study on a larger set of songs and punctuators will be necessary to confirm its findings."

Response to reviewer 2:

Original comment: *i) It is not clear for me the role of Coh-Metrix in the paper. Is it used only for counting sentences? There is a whole corpus of introduction mentioning the advantages and the importance of pre-processing before the use of Coh-Metrix, but at line 317 the authors say that their analysis focus only on the number of sentences, instead of "more sophisticated Coh-Metrix measures" (line 318).*

The question as to the relevance of the sentence is a central one. Sentence count is itself a measure, but more importantly, it is an underlying contributor to a large range of more sophisticated measures. As such, we have investigated the differences in sentence count between three different punctuators and draw the conclusion that more sophisticated measures relying on sentence count as a contributing measure would be affected by significant differences in sentence count (such as word overlap between sentences, semantic overlap between sentences, latent semantic analysis, to name a few). This is explained in the section "The relevance of the sentence in Coh-Metrix 3.0".

The section includes the following explanation: Quote from lines 46-68: "It can be speculated that lexical information such as diversity measures, word frequency, word meaningfulness and concreteness, would be largely unaffected by sentence segmentation, whereas certain syntactic indices such as sentence syntax similarity of adjacent sentences (SYNSTRUTa) clearly rely on the sentence as a unit.

Similarly, cohesion measures such as noun-, argument-, stem-, and content-word overlap in adjacent sentences or across all sentences in a text require sentence boundaries to be indicated, as do measures of Latent Semantic Analysis (LSA), that is, measures of relative semantic overlap between sentences and paragraphs.

The same holds for traditional readability scores included in Coh-Metrix 3.0, the Flesch Reading Ease (RDFRE) and the Flesch-Kincaid Grade Level (READFKGL), which both rely on sentence length (mean number of words per sentence) to compute text difficulty.

The Coh-Metrix L2 Readability score (L2), intended to assess the suitability of texts for second language learners, includes content-word overlap in and syntactic similarity of sentences as variables in its formula.

Finally, Coh-Metrix 3.0 offers so-called Text Easability Principal Component Scores, based on a principal components analysis (PCA) of 54 indices in a corpus of written texts found across school-grade levels (K-12) and academic subjects (see Graesser (2011), McNamara(2014) for further details). Five of these principal components – narrativity, syntactic simplicity, word concreteness, referential cohesion, and deep cohesion, account for 54% of the variance in the test corpus and are provided to Coh-Metrix users as comprehensive scores that are more easily accessible and of more immediate practical use for teaching practitioners.

Graesser (2011) show that sentence-based measures contribute to three of these easability scores (narrativity, syntactic simplicity, referential cohesion)."

Furthermore, the argument of the sentence as an underlying measure contributing to a range of other measures is emphasised in our Discussion so that it connects with the following quote:

Quote from lines 406-415: "We have, thus, demonstrated on a small scale that differences in how a text is pre-processed can lead to a significant difference in the number of sentences. This has implications for at least some Coh-Metrix measures such as sentence length/mean number of words (DESSL) or paragraph length/mean number of sentences (DESPL). As these descriptive measures significantly contribute to a range of other, more sophisticated measures, it can be assumed that these might be affected as well. Consequently, it seems that normalisation of non-traditional written texts that do not clearly indicate sentence boundaries should receive greater attention during planning, analysis and documentation."

Original comment: *ii) In line 329, it is said "Note that we have overdispersion in the counts". The reader has some trouble to follow this line of thought because we do not have access to the distributions of the number of counts. I would suggest presenting this distribution or, for instance, the inclusion of a figure with the song id in the x-axis (from 1 to 30) and the number of sentences in the y-axis: this figure would have 3 curves, each one indicating the number of sentences obtained from the punctuation of each transcriber. In this example, the dispersion of the data could be seen in the range of y.*

Well spotted, this was certainly something we needed to explain better in order for the reader to not just take our word for it, but be able to see it for themselves. Thank you for the helpful suggestion for a plot which would show the overdispersion. However, a plot takes up a lot of space, and we weren't sure if the reader would know what to look for (as in, we would need to give some explanation as well) so we instead decided to do test for overdispersion as another way of getting the reader to understand how we detect overdispersion.

That is, in section 5, as highlighted, we explain the idea of the test, e.g. that the test statistic is the ratio of the expected variance (under the model) and the observed variance. We then use the highly significant p-value as proof that we should consider a quasi-Poisson model instead to account for the difference in the true variance from the variance assumed under the model.

Original comment: *iii) The authors should be more descriptive about the tests realized. I believe section 5 could be expanded for the sake of better comprehension. For instance, although ANOVA tables are pretty common, it would be nice to guide the reader to how it is obtained (in a few words) and to the meaning of each column presented in table 2, as well as the relevance of the results to the purpose of the paper.*

Once again, following this advice will improve the article greatly, and as such we have added clarification in section 5 as highlighted.

Original comment: *iv) The authors could explain why they decided to use the Poisson generalized linear model. I mean, is it expected the number of sentences (NoSen) to follow a Poisson distribution?*

This is correct, we do indeed treat the number of sentences as counts, and thus assume they are Poisson distributed. Clearly, this was not explained well enough in the current form of the article and thus the explanation has now been given more attention when addressing the previous comment.

Original comment: *In conclusion, it is expected that changes in text segmentation would result in differences on text metrics such as the number of sentences. In my opinion, the most interesting aspect of the paper is the quantification of the different interpretations of the same songs: as pointed out in subsection 4.2.1, the transcribers were instructed to follow their "gut feeling" (line 293). In this sense, the results measure how the same set of song can be perceived, felt, seen, viewed, by the transcribers. The importance of this point is already mentioned by the authors when they talk about the ESL (lines 16, 450), but I would suggest an emphasis in the aspect of "quantification of the differences in perception", including the title and abstract, to make the paper stronger.*

Our research shows that different transcribers delineate the structural unit of the sentence differently. However, our research does not provide insight into whether these decisions are made based on semantic interpretation (felt, seen, viewed) or solely on different views on the definition of the sentence unit, on sentence grammar, acceptability and style. It can be assumed that it is a combination of these factors, but that assumption cannot be inferred from the present results.

What our study shows is that there can be differences in text segmentation between educated transcribers and these differences can have an impact on the results of automated quantification of textual features. Based on a review of quantitative studies involving Coh-Metrix and also literature describing the role of the sentence in NLP, we can also state that this problem does not seem to have received any attention.

Based on your thoughtful feedback we have updated the title to "The impact **of differences in** text segmentation on **the** automated **quantitative** evaluation of song-lyrics" as well as the abstract as highlighted in the revised manuscript.

Again, thanks so much for your input!

Sincerely,

Friederike and Katharina
